# Modeling Cerebral Blood Flow Dependence on Carbon Dioxide and Mean Arterial Blood Pressure in the Immature Brain With Accounting for the Germinal Matrix

**DOI:** 10.3389/fneur.2018.00812

**Published:** 2018-10-09

**Authors:** Irina Sidorenko, Varvara Turova, Nikolai Botkin, Laura Eckardt, Ana Alves-Pinto, Ursula Felderhoff-Müser, Esther Rieger-Fackeldey, Andrey Kovtanyuk, Renée Lampe

**Affiliations:** ^1^Mathematical Faculty, Technical University of Munich, Garching, Germany; ^2^Orthopedic Department, University Hospital Rechts der Isar, Technical University of Munich, Munich, Germany; ^3^Pediatric Department I, Neonatology, Pediatric Intensive Care, Pediatric Neurology, University Hospital Essen, University Duisburg-Essen, Essen, Germany; ^4^Pediatric Department, University Hospital Rechts der Isar, Technical University of Munich, Munich, Germany

**Keywords:** intraventricular hemorrhage, germinal matrix, cerebral autoregulation, gestational age, capillary wall tension

## Abstract

Intraventricular hemorrhage (*IVH*) is one of the most critical complications in the development of preterm infants. The likelihood of *IVH* is strongly associated with disturbances in cerebral blood flow (*CBF*) and with microvascular fragility in the germinal matrix (*GM*). The *CBF* value and its reactivity to changes in arterial carbon dioxide pressure (*pCO*_*2*_) and mean arterial blood pressure (*MABP*) are relevant indicators in the clinical assessment of preterm infants. The objective of the present study is mathematical modeling of the influence of *pCO*_*2*_ and *MABP* on *CBF* in immature brain, based on clinical data collected from 265 preterm infants with 23–30 gestational weeks. The model was adapted to the peculiarities of immature brain by taking into account the morphological characteristics of the *GM* capillary network and vascular reactivity, according to gestational and postnatal age. An analysis of model based values of *CBF* and its reactivity to changes in *MABP* and *pCO*_*2*_ was performed separately for each gestational week and for the first two days of life both for preterm infants with and without *IVH*. The developed model for the estimation of *CBF* was validated against equivalent experimental measurements taken from the literature. A good agreement between the estimated values of *CBF*, as well as its reaction on changes in *MABP* and *pCO*_*2*_ and the equivalent values obtained in experimental studies was shown.

## Introduction

Advances in neonatal care have increased the chances of survival of preterm infants, but the occurrence of complications in postnatal development remains high. One of the most frequent complications, around 15–20% of cases, in infants born before 32 weeks of gestation (*WG*) is intraventricular hemorrhage (*IVH*) (1), which can lead to lifelong impairments such as cerebral palsy. Hemorrhages occur mostly in the subependymal germinal matrix (*GM*) (2), a specific region in the immature brain located between the thalamus and caudate nucleus, having a high vascularity (3) and a fragile capillary network. The *GM* reaches its maximum size of 5% from the total brain volume at 22 *WG*, rapidly shrinking after 28 *WG* and practically disappearing by 34 *WG* (3). Electron-microscope observations have shown that the density of micro vessels in this region is about 1.5 times higher than in any other part of the brain and that the average diameter of vessels is also larger than in the cortex (4, 5). According to Laplace's law, vessels in the *GM* are, therefore, subjected to larger wall tensions and, consequently, they have higher probability of rupture (5). There is also evidence that, due to the lack of collagen and elastane, the walls of *GM* vessels are thinner (5), have less supporting tissue, and are more fragile than in other parts of the brain. This makes vessels of the *GM* more sensitive to fluctuations in *CBF* than vessels in other regions of the brain. Therefore, the assessment of physical properties of the microcirculation from standard medical parameters is important for the evaluation of biomechanical stability of capillaries in *GM*.

Another pathological mechanism that can increase the likelihood of intracranial hemorrhage in preterm infants is a weak autoregulatory response of arterioles to changes in mean arterial blood pressure (*MABP)* and in partial pressure of blood gases (6–8). An intact autoregulatory mechanism keeps cerebral blood flow (*CBF)* almost constant despite changes in *MABP*. Impaired autoregulation leads to a linear dependence of *CBF* on *MABP* (7) (designated as “passive autoregulation” below). A high fragility of vessel walls in the *GM* and a deficient cerebral autoregulation in the immature brain make it more sensitive to fluctuations in *CBF* than the mature brain of full-term infants (4, 9), which can contribute to trigger a hemorrhage and/or ischemia.

Hence, regular monitoring of the *CBF* level as well as observing its fluctuations caused by passive autoregulation are critical to prevent the occurrence of complications in preterm infants. Several techniques for measuring *CBF*, such as near-infrared spectroscopy (10–12) (NIRS), Xenon-133 clearance measurements (10, 13, 14), transcranial Doppler ultrasonography (9, 15), MRI based arterial spin labeling (16, 17) (MRI ASL) and diffusion correlation spectroscopy (12, 18–20) (DCS), are currently available in medical treatment. However, they are not yet part of clinical routine. An alternative approach is to employ mathematical models of cerebral hemodynamics (21, 22) to estimate *CBF*. During recent years, numerical simulations describing the origin, influencing factors, and consequences of intraventricular hemorrhage in preterm infants have been developed (23, 24). These simulations, however, did not take into account the presence of the *GM* in the premature brain.

The purposes of the present study were, therefore, the following: (I) Development of a mathematical model for numerical evaluation of *CBF*, taking into account a realistic description of the *GM* along with cerebral autoregulation, according to the gestational age of each patient. (II) Demonstration of the research capabilities of this model in assessing physical properties of the microcirculation in the *GM*. (III) Verification of the model by comparison of model based values of *CBF*, as well as *CBF* reactivity to changes in *MABP* and *pCO*_2_, with equivalent measurements from experimental studies reported in the literature (9–14, 17).

## Materials and methods

### Description of the sample group

Clinical data were obtained retrospectively from the records of 265 preterm infants treated in the Departments of Neonatology of the University Hospital Rechts der Isar of the Technical University of Munich (MRI TUM) and of the University Hospital of Essen. The project was approved by the Ethics Committees of the University Hospital Rechts der Isar, Technical University of Munich (Ref. 364/15) and of the University Hospital Essen, University Duisburg-Essen (Ref. 16-7284-BO).

The gestational age of the sample group ranged from 23 to 30 *WG* (26.1 ± 2.2 *WG*), and the birth weight from 335 to 1580 g (856.66 ± 278.94 g). Although all infants included in this study were preterm, we divided them into control and risk groups according to the absence or presence of *IVH*. The cranial ultrasound examinations were performed routinely on the 1st, 3rd, 7th, 14th days and sometimes more often (even daily) in case of abnormalities or suspected bleeding. *IVH* was diagnosed in 136 cases (risk group). The remaining 129 patients were not diagnosed with *IVH* (control group). Separation of patients into groups according to *IVH* degree was not done here, because this difference is not accounted for in the present mathematical model. Obstetric characteristics for different gestational ages are presented in Tables 1, 2. A total of 107 (78.7%) infants were diagnosed with *IVH* during first 5 days, which is typically caused by hemodynamic instability. Another 29 (21.3%) infants had *IVH* after day 5, which may indicate that other clinical factors contributed to origin of bleeding. Available clinical details for 29 infants, for whom *IVH* was diagnosed after day 5, were reviewed to decide whether or not those infants should be included in the analysis (see Table 3). In percentage, there were more cases of EPH Gestosis, Necrotizing Enterocolitis, Thrombocytopenia, Cardiopulmonary Adaptation, and Cholestasis in infants with *IVH* diagnosis after day 5 than in infants with *IVH* diagnosed within the first 5 days after birth. However, since there is no direct evidence for the presence or absence of a causal influence of these factors on hemodynamic stability, the 29 infants were included into the analysis.

**Table 1:** Obstetric characteristics of the control (no *IVH*) and risk (with *IVH*) groups.

**WG**	***IVH***	**Number**	**Male**	**Twins**	**Triplets**	**Birth weight [g]**
23	No	12	2	2	2	520.83 ± 66.80
	With	17	5	3	6	531.18 ± 79.78
24	No	26	9	6	1	615.58 ± 103.70
	With	24	14	9	2	657.71 ± 125.33
25	No	19	10	0	2	688.94 ± 77.27
	With	23	9	7	3	750.87 ± 100.79
26	No	17	5	0	2	766.43 ± 68.71
	With	20	16	9	0	822.00 ± 136.91
27	No	13	6	2	4	902.31 ± 109.78
	With	15	8	4	1	948.33 ± 226.45
28	No	16	7	5	4	1017.2 ± 76.11
	With	18	11	3	0	1157.5 ± 168.16
29	No	13	5	6	1	1156.9 ± 67.09
	With	11	6	1	1	1259.5 ± 213.76
30	No	13	9	5	1	1274.6 ± 83.33
	With	8	5	1	2	1456.2 ± 115.01
All	No	129	53	26	17	838.59 ± 253.79
	With	136	74	37	15	875.71 ± 300.51
All	All	265	127	63	32	856.66 ± 278.94

**Table 2:** The day of *IVH* diagnosis (number of diagnosed infants for each gestational week).

**WG**	**Total number**	**on 1st day**	**on 2*nd* day**	**on 3rd day**	**on 4th day**	**on 5th day**	**after 5th day**
23	17	3	2	7	1	1	3
24	24	3	3	9	6		3
25	23	3	4	7	5	1	3
26	20		3	10	2	3	2
27	15	4	1	1	3	2	4
28	18	2	5	4	1	1	5
29	11	1	1	2	2	2	3
30	8		1			1	6
All	136	16	20	40	20	11	29

**Table 3:** Baseline clinical parameters for infants with *IVH* before and after 5th postnatal day.

	***IVH* ≤ 5 days**	***IVH* > 5days**
Number of infants with *IVH*	107 (100%)	29 (100%)
Twins	29 (27.1%)	8 (27.6%)
Triplets	11 (10.3%)	4 (13.8%)
CHARGE	0	1 (3.4%)
Esophageal Atresia	0	1 (3.4%)
Intrauterine Growth Retardation (IUGR)	4 (3.7%)	2 (6.9%)
Feto-Fetal Transfusion Syndrome (FFTS)	3 (2.8%)	1 (3.4%)
EPH Gestosis	3 (2.8%)	4 (13.8%)
*In Vitro* Fertilization (IVF)	12 (11.2%)	3 (10.3%)
Lung Bleeding	13 (12.2%)	3 (10.3%)
Neonatal Bowel Perforation	8 (7.5%)	3 (10.3%)
Necrotizing Enterocolitis (NEC)	8 (7.5%)	4 (13.8%)
Disseminated Intravascular Coagulation (DIC)	0	2 (6.9%)
Thrombocytopenia	7 (6.5%)	5 (17.2%)
Cardiopulmonary Adaptation	4 (3.7%)	4 (13.8%)
Cholestasis	5 (4.7%)	4 (13.8%)
Pulmonary Stenosis	0	1 (3.4%)
Intubation	89 (83.2%)	25 (86.2%)
Sepsis	56 (52.3%)	14 (48.3%)
Death	25 (23.4%)	3 (10.3%)

To account for the fast involution (3) of the *GM* after 22 *WG* (Table 4) and impaired autoregulation during first days of postnatal life (13), patients were assigned to groups according to the gestational age in weeks, and data were analyzed for different postnatal days.

**Table 4:** Volume fraction of germinal matrix (in% from total brain volume) (3).

***WG***	**23**	**24**	**25**	**26**	**27**	**28**	**29**	**30**	**40**
*GM*%	5	3.3	2.3	1.7	1	0.7	0.3	0.1	0

### Collection of clinical data

The *MABP* and *pCO*_*2*_ records were collected from standard clinical measurements during routine clinical nursing for the first 10 days after birth in the control group, and for up to 7 consecutive days before and 3 days after hemorrhage in the risk group. Only arterial and capillary blood gas values were considered in the analysis. *MABP* was measured either invasively via an arterial catheter or non-invasively with a cuff placed on the upper part of the neonate's arm. No distinction between two measuring methods was done because of a good agreement between them, irrespective of birth weight and gestational age of preterm infants (25). Although measurement of *MABP* and *pCO*_*2*_ were done at different times, only coincident records of *MABP* and *pCO*_*2*_ were used in numerical computations. The number of coincident records of *MABP* and *pCO*_*2*_ varied from 1 to 44 per patient, with a median of 12 and an interquartile range of 10. The total number of coincident records was 3439.

### Statistical methods

Statistical description of clinical records was required for modeling the cerebral autoregulation processes in preterm infants. Mean values of *MABP* and *pCO*_*2*_ were estimated from the control group and taken as reference values in the mathematical model of cerebral autoregulation.

Fast changes in the *GM* size (3) and vessels' reactivity (11, 13) during the development suggest a correlation between the *CBF* and gestational age (in weeks) as well as postnatal age (in days). Therefore, a statistical analysis of clinically measured *MABP* and *pCO*_*2*_, model based *CBF*, and its sensitivity to *MABP* and *pCO*_*2*_ was done separately for each gestational week and for the first two postnatal days.

Statistical comparisons of relevant variables between infants with and without *IVH* were also performed. Comparisons were done using either the Student *t*-test or the Wilcoxon rank-sum test, depending on whether data were normally distributed or not, which was controlled with Kolmogorov-Smirnov tests. All statistical analyses were carried out with the standard statistical library of MATLAB R2018a.

### Mathematical model of cerebral blood vessel system

**Figure 1:**
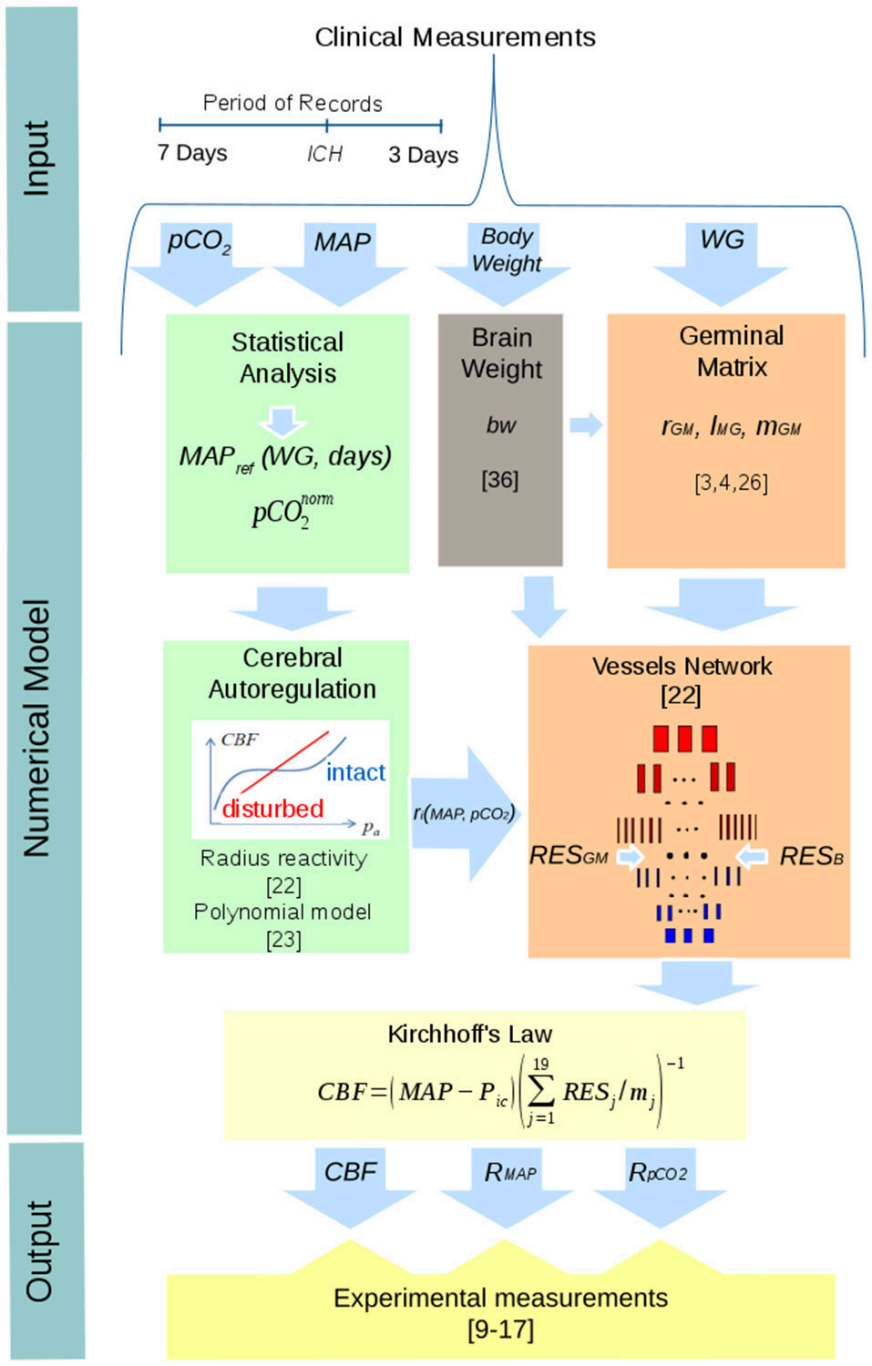
Schematic representation of the numerical model of *CBF* used in this study and of the procedure used in its validation.

A mathematical model of adult brain (22) was adapted to describe the cerebral hemodynamics of immature brain in preterm infants. The following adaptations to the model were done: (I) The number of vessels as well as their lengths and diameters were scaled down in agreement with experimental measurements (4, 26) and according to the brain weight of each infant. (II) The presence and extension of the *GM* was simulated according to gestational age (3). (III) A phenomenological cerebral autoregulation model (23) describing the myogenic response to fluctuations in *MABP* was incorporated into the hierarchical cerebrovascular model (22) and adjusted to preterm infants, based on average values derived from the clinical records of preterm infants without *IVH*. (IV) Vascular activity was simulated as changes in the vessel radius in response to changes in *MABP* and carbon dioxide pressure (*pCO*_*2*_), with accounting for gestational and postnatal age. Since carbon dioxide is 20 times more soluble in blood than oxygen, and *CBF* is almost 10 times more sensitive to changes in *pCO*_*2*_ [reactivity (9) 32.7% kPa^−1^] than in *pO*_2_ [reactivity (9) −3.1% kPa^−1^], carbon dioxide is recognized as a major determinant of *CBF* (27). Therefore, the effect of *pO*_2_ was neglected in the mathematical modeling of *CBF*. (V) The presence of red blood cells was accounted for by application of a micropolar fluid model (28–30) of blood flow (24). The methods and adaptations performed are schematically described in Figure 1.

Numerical calculation of the *CBF* was based on a hierarchical cerebrovascular model proposed for adult brain (22) and adjusted to the case of preterm infants (23, 24). In this approach, the vascular system is divided in 19 levels according to the morphological characteristics of the vessels. The magnitude of *CBF* is derived from the Kirchhoff's law as:

$$\begin{array}{l} CBF=\left(MABP-P_{ic}\right){\left(\sum_{j=1}^{19}RES_{j}^{layer}\right)}^{-1}\\ =\left(MABP-P_{ic}\right){\left(\overset{19}{\underset{j=1}{\sum }}RES_{j}/m_{j}\right)}^{-1}, \end{array}$$

where *MABP* is the mean arterial blood pressure, *P*_*ic*_is the intracranial pressure (*ICP*), *m*_*j*_ and *RES*_*j*_ are the number and resistance of vessels on level *j*, respectively. In numerical calculations *MABP* is taken from the clinical records of each patient. The *P*_*ic*_value is also individual for each patient and could be inserted into the mathematical model directly from clinical records. However, because of missing *ICP* records in our data, a constant *P*_*ic*_ value of 5 mmHg was assigned to all patients. The mean *ICP* values in preterm neonates can vary from 2.7 mmHg (31, 32) to 12.5 mmHg (33) depending on the measurement method. However, according to (32), *ICP* values greater than 7 mmHg are “indicative of intracranial hypertension, but occurred in relation to nursing events and positioning of the patient's head.” The used *ICP* value of 5 mmHg is a mean between 3 and 7 mmHg, which is in accordance with the mean *ICP* values of 4–6.5 mmHg (34) and 5.1 mmHg (35) measured in preterm infants.

Thus, the volume of blood flow depends on the perfusion pressure, *MABP*−*P*_*ic*_, and vessel resistances, *RES*_*j*_, influenced by the density and morphology of the vessel network and the rheological characteristics of blood. In the simplest case of Hagen-Poiseuille's flow of Newtonian fluid, the vessel resistance *RES*_*j*_ is given by:

$$\begin{array}{l} RES_{j}=8\mu l_{j}/\pi r_{j}^{4}, \end{array}$$

where μ is the dynamic viscosity of blood, and *l*_*j*_ and *r*_*j*_ are the length and radius of vessels on each level *j* = *1 … 19*. The vessel radius *r*_*j*_ is not constant but varies depending on the reactivity of blood vessels (i.e., vasodilation or vasoconstriction) to *pCO*_*2*_ and *MABP* (see section Accounting for Cerebral Autoregulation below).

In the present work, the setting $RES_{j}=RES_{j}^{mp}$, where the right-hand side was derived using micropolar description (28–30) of blood flow, is utilized. This technique accounts for the presence of rigid, randomly oriented particles (red blood cells) suspended in a viscous medium. The method assumes the introduction of additional rotational degrees of freedom into the flow equations. In the case of incompressible steady state flow through a pipe with circular section, a power series expansion of solutions yields an easily computable approximation of the flow velocity and flow resistance under the assumption of constant hematocrit (24, p. 5–8). We do not show here the corresponding formulas obtained with the Maple software for symbolic computations because they are exceedingly long.

The modification of the number of vessels *m*_*j*_, their initial diameter $d_{j}^{0},$ and length *l*_*j*_, on each hierarchical level *j, j* = *1 … 19*, in comparison with these values for adult brain (22), was done according to the gestational age and brain weight *bw* under the following empirical assumptions: (I) The number of main arteries (*m*_1_*)* and veins (*m*_19_) is constant across age. (II) The number of small arterioles, capillaries, and venules decreases depending on the amount of brain tissue. (III) The length and the diameter of large arteries and veins decrease with brain weight. (IV) The morphometric values of capillaries are similar to the values used in the model of adult cerebral microvascular system (22). Such definition of vessel size is supported by studies (26) suggesting that characteristics of the capillary network are determined by metabolic processes and are similar across mammals. Thereby, the following modifications in the number of vessels *m*_*j*_, their length *l*_*j*_, and diameter *d*_*j*_ were applied:

$$\begin{array}{l} m_{j}=M_{j}\cdot {\left(1200/bw-\left(1200/bw-1\right)\cdot |j-10|/9\right)}^{-1},\\ l_{j}=L_{j}\cdot {\left(1-0.1\cdot \left(1200/bw-1\right)\cdot |j-10|/9\right)}^{-1},\\ d_{j}=D_{j}\cdot {\left(1-0.1\cdot \left(1200/bw-1\right)\cdot |j-10|/9\right)}^{-1}. \end{array}$$

Here *M*_*j*_, *L*_*j*_ and *D*_*j*_ are the number, length and diameter of vessels in level *j* of the adult brain, respectively. The coefficient 0.1 is used to scale the vessel length and diameter to experimental measurements (4, 26). The value of 1,200 g corresponds to the approximate weight of the adult brain (22) and *bw* is the brain weight of the preterm infant, computed from his body weight (*BW*) according to the regression formula (36):

$$\begin{array}{l} bw\left(BW\right)=-3.09+0.15BW-1.064\cdot 10^{-5}BW^{2}. \end{array}$$

### Accounting for the presence of the germinal matrix

The model network used in this study included an additional part, at the capillary level, describing the presence of the *GM* in the brain of infants with *WG* < 32. Accounting for the *GM* in the computation of *CBF* was implemented through dividing the vascular system at the capillary level, *j* = 10, into two parallel circuits. One circuit describes the *GM* with the total hydraulic resistance $RES_{GM}^{mp}$ calculated with the above mentioned micropolar flow model (24) and with the number of (parallel connected) vessels given by:

$$\begin{array}{l} m_{GM}=M_{10}/\left(1200/bw\right)\cdot GM_{vf}\cdot 1.5. \end{array}$$

Here *M*_10_ is the number of capillaries on the 10th level of the adult vascular network (22), *GM*_*vf*_ is the fractional volume of the *GM* relative to the total brain volume (3) (see Table 4), and the factor 1.5 describes a density correction factor (4) for the *GM*.

The second circuit corresponds to the rest of the capillary system of the brain. Its total hydraulic resistance $RES_{B}^{mp}$ was calculated using the number of (parallel connected) vessels given by:

$$\begin{array}{l} m_{B}=M_{10}/\left(1200/bw\right)\cdot \left(1-GM_{vf}\right). \end{array}$$

Values of capillary length and diameter were taken from the literature as follows: *l*_*GM*_ = 40 μm, *d*_*GM*_ = 6.7 μm for the *GM* (4, 26), and *l*_*B*_ = 60 μm and *d*_*B*_ = 5.6 μm for the rest of the brain (22).

The total resistance of the capillary level becomes

$$\begin{array}{l} RES_{10}^{level}={\left({\left(RES_{GM}^{mp}\right)}^{-1}+{\left(RES_{B}^{mp}\right)}^{-1}\right)}^{-1}. \end{array}$$

It is important to note that the effect of the *GM* on the global *CBF* is rather small because of the relatively small volume of *GM*. However, because of the larger radius of the *GM* capillaries, the wall tension is likely to be larger than that in the capillaries of other brain regions. Notice that the wall tension in capillary vessels obeys Laplace's law:

$$\begin{array}{l} T=P_{10}\cdot r/t, \end{array}$$

where *P*_10_is the pressure in the capillary level, *t* = 0.5 μm is the thickness of the capillary wall, and *r* is the radius of the capillary, in the *GM* or in the other region of the brain, altered (vasodilation or vasoconstriction) as a result of changes in *pCO*_*2*_ and *MABP*.

### Accounting for cerebral autoregulation

Vessel reactivity plays a major role in supporting a stable *CBF*. Fluctuations in *MABP* and *pCO*_*2*_ induce contraction and relaxation of muscle cells within vessel's walls. Vasodilation and vasoconstriction change vessel's radius and hence vessel's resistance to blood flow. Autoregulation processes mainly occur in arteries and arterioles. Capillary walls do not have layers with muscular tissue, but they also elastically react to fluctuations in *CBF*. Moreover, the role of pericytes in regulating capillary diameter was recently addressed (37).

In the hierarchical cerebrovascular model (22) the influence of *pCO*_*2*_ on *CBF* is modeled through the modification of the vessel radius as follows, where *c*_*j*_ are the reactivity coefficients:

$$\begin{array}{l} r_{j}^{pCO_{2}}=r_{j}^{o}\cdot \left(1+c_{j}\cdot \left(pCO_{2}-pCO_{2}^{norm}\right)\right). \end{array}$$

The reactivity coefficients *c*_*j*_ are different for different vascular levels (22). The strongest reaction with *c*_8_ = 13.9% kPa^−1^ (1.81% mmHg^−1^) is demonstrated by arterioles at the 8th level. For capillaries and venules it decreases reaching the lowest value of *c*_19_ = 2.1% kPa^−1^ (0.28% mmHg^−1^) for veins at the 19th level.

The increase/decrease in the vessel radius *r*_*j*_ is a function of the deviation of *pCO*_*2*_ from the baseline value $pCO_{2}^{norm}$. In the present work, we take $pCO_{2}^{norm}\;=$ 40 mmHg, which is the middle value between the lower and upper bounds of normocapnia described in the literature as 35 ≤ *pCO*_*2*_ ≤ 45 mmHg (38) or 30 ≤ *pCO*_*2*_ ≤ 55 mmHg (39).

Experimental measurements of the *CBF* reactivity to *pCO*_*2*_ in preterm infants showed that the reactivity was decreased down to 10% kPa^−1^ (1.3% mmHg^−1^) (15, 27) on the first day of life but could reach normal values of 30% kPa^−1^ (4% mmHg^−1^) (16, 22) already on the second day. This reduction in the reactivity on the first day of life was accounted for in the simulations by decreasing the reactivity coefficients *c*_*j*_ by a factor of 3 for the first day of life.

Another very important mechanism of cerebral autoregulation is the myogenic response to fluctuations in blood pressure. To simulate this mechanism, the cerebrovascular model was extended by adding the effect of *MABP* on the vessel radius as follows (23):

$$\begin{array}{l} r_{j}\left(MABP,pCO_{2}\right)=r_{j}^{pCO_{2}}\cdot {\left[\left(MABP_{ref}-P_{ic}\right)/\left(MABP-P_{ic}\right)\right]}^{1/4}\\ \cdot [1+a_{1}\cdot \left(MABP-MABP_{ref}\right)+a_{2}\cdot {\left(MABP-MABP_{ref}\right)}^{2}\\ \;+\;a_{3}\cdot {\left(MABP-MABP_{ref}\right)}^{3}] \end{array}$$

**Figure 2:**
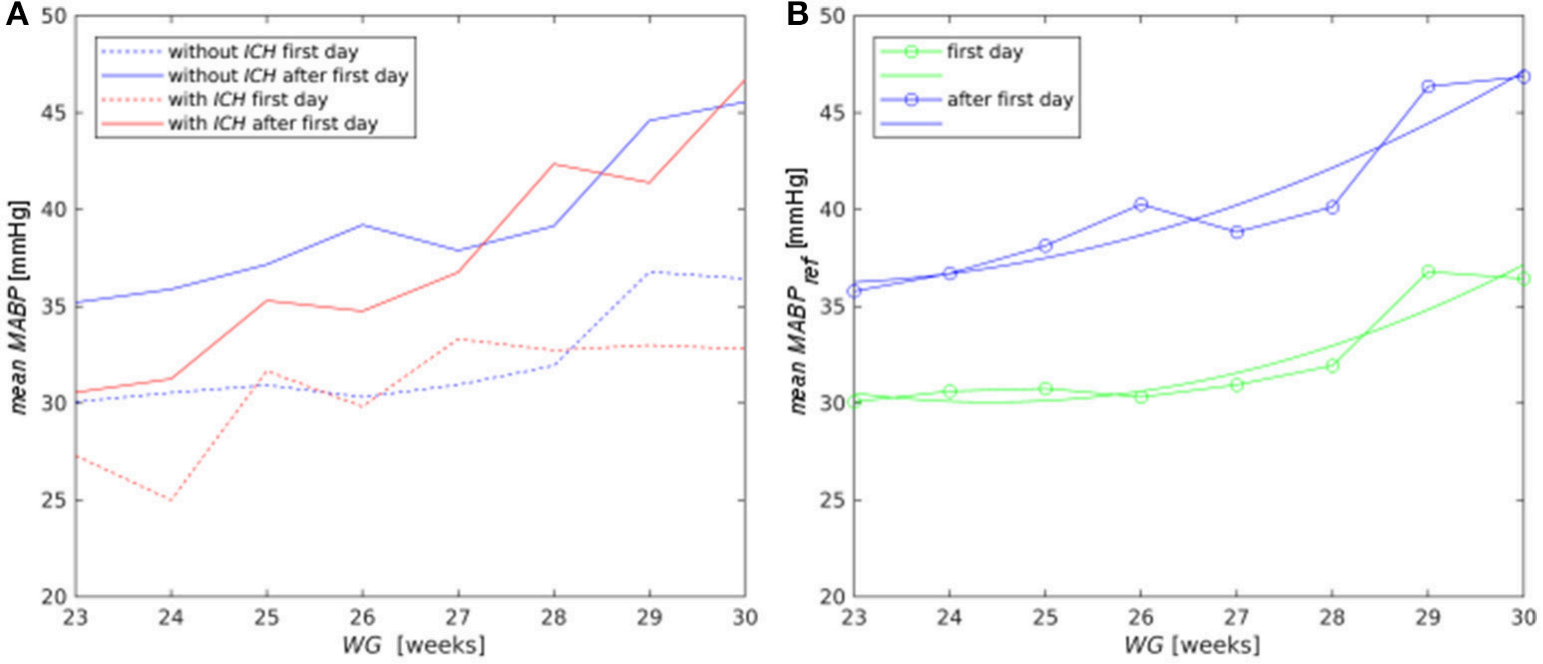
**(A)** Mean *MABP* for the control (blue lines) and risk (red lines) groups for the first day (dashed lines) and for the following days (solid lines); **(B)** polynomial approximation (lines without symbols) to the average pressure *MABP*_*ref*_ obtained from the clinical records (lines with symbols) in the control group for the first day (green curves) and for the period after the first postnatal day (blue curves).

For a fixed *pCO*_2_, the dependence of *r*_*j*_on *MABP* has a sigmoidal shape with a plateau around the reference pressure *MABP*_*ref*_. This dependence was adjusted to experimental data via the polynomial coefficients *a*_*i*_ (23). The value of *MABP*_*ref*_ and the width of the plateau are much smaller in newborns than in adults (30). However, *MABP*_*ref*_ increases with gestational and postnatal age (see Figure 2A). This behavior was accounted for by using polynomial fits to estimate *MABP*_*ref*_ as function of *WG* for each postnatal day. The following results, shown also in Figure 2B, were obtained with a standard library of MATLAB R2018a:

$$\begin{array}{l} MABP_{ref}^{day=1}\left(WG\right)=62.24-3.112\cdot WG+0.07396\cdot WG^{2},\\ MABP_{ref}^{day=2}\left(WG\right)=-47.39+4.87\cdot WG-0.06328\cdot WG^{2},\\ MABP_{ref}^{day\geq 2}\left(WG\right)=-49.99+5.397\cdot WG-0.07441\cdot WG^{2}. \end{array}$$

When analyzing the combined effect of *MABP* and *pCO*_*2*_, both the risk of hemorrhage and ischemia need to be considered. If vessels are dilated at *pCO*_*2*_ > 50 mmHg, additional vessel dilation due to decreasing *MABP* can lead to *IVH*. If *pCO*_*2*_ < 30 mmHg, and vessels are constricted, additional vessel constriction as a reaction to increasing *MABP* can lead to ischemia (40, 41). These bounding values (30 and 50 mmHg) provide physiologically acceptable limits for intact autoregulation. Thus, the vital range for *pCO*_*2*_ ensuring stable *CBF* was assumed to be |*pCO*_*2*_– ${\mathrm{pCO}}_{2}^{norm}$| ≤ 10 mmHg (41) with $pCO_{2}^{norm}=$ 40 mmHg (38, 39). As for *MABP*, the intact autoregulation was defined by the condition |*MABP – MABP*_*ref*_| ≤ 5 mmHg (6, 40, 41).

**Figure 3:**
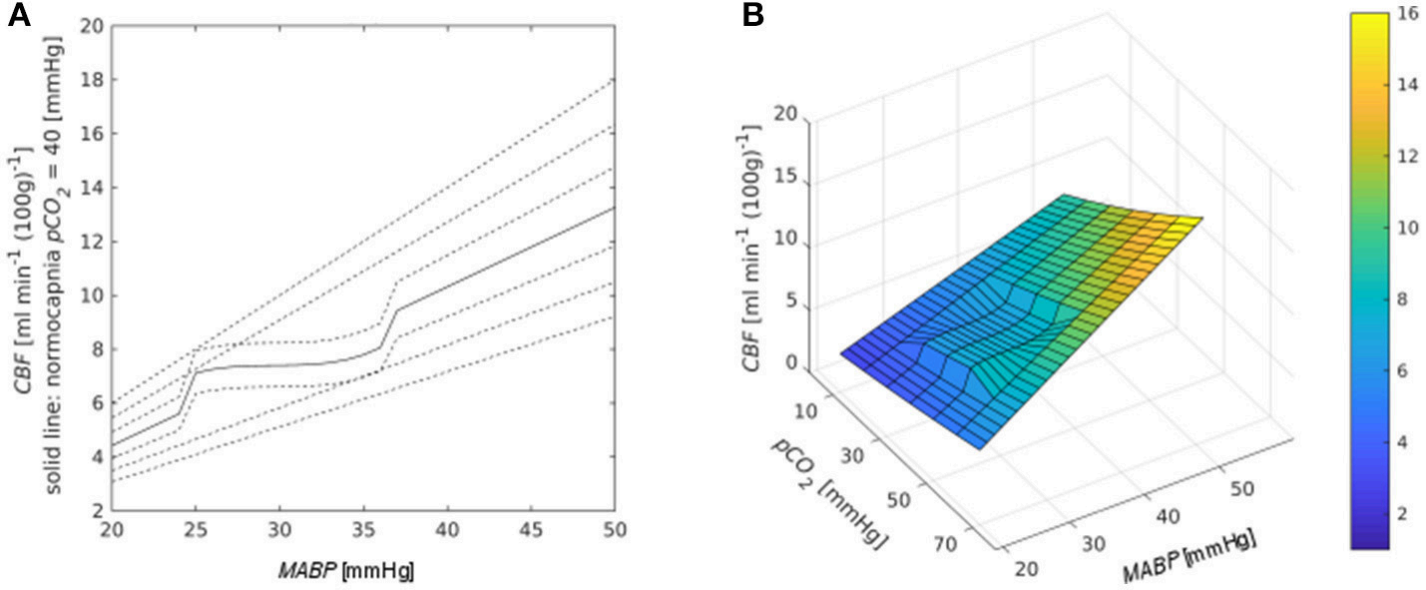
**(A)** Dependence of *CBF* on *MABP*. The platea occurs around *MABP*_*ref*_ = 30 mmHg. Different lines correspond to different values of *pCO*_*2*_ in mmHg [from bottom to top: 10, 20, 30, 40 (solid line corresponds to normocapnia), 50, 60, 70]. **(B)** Combined effect of *MABP* and *pCO*_*2*_ on *CBF*.

The modeled effect of *MABP* on *CBF* for different *pCO*_*2*_ values is illustrated in Figure 3A for 23 *WG*. The autoregulatory plateau in Figure 3A corresponds to the region of stable *CBF*, formed around the reference pressure *MABP*_*ref*_ = 30 mmHg. The plateau disappears for *MABP* < 25 mmHg and *MABP* > 35 mmHg. Outside the plateau *CBF* changes passively in a linear way with respect to *MABP*. Figure 3B illustrates the combined effect of *MABP* and *pCO*_*2*_ on *CBF*, with the reference pressure value at *MABP*_*ref*_ = 30 mmHg and ${\mathrm{pCO}}_{2}^{norm}$ = 40 mmHg. The autoregulatory plateau corresponding to stable *CBF* is located in the region {25 mmHg < *MABP* < 35 mmHg, 30 mmHg < *pCO*_*2*_ < 50 mmHg}. In all other regions, *CBF* depends linearly on *MABP* and *pCO*_*2*_. The absence of the autoregulatory plateau and linear dependence of *CBF* on *MABP* for *pCO*_*2*_ > 50 mmHg in our model is in agreement with the autoregulation breakpoint *pCO*_*2*_ = 51 mmHg identified with bilinear regression analysis of Doppler measurements of *CBF* (15).

## Results

### Analysis of clinical data

Mean *MABP* increased both with gestational and postnatal age (Figure 2A). On the first day, the mean *MABP* of the risk group was lower than that of the control group only for infants with WG < 25 (*p* = 0.002). However, from the second day on, infants from the risk group with *WG* < 28 had lower *MABP* than infants from control group (*p* = 0.057). The mean *MABP* value of the control group was used as the reference pressure *MABP*_*ref*_ (Figure 2B) in the autoregulation law of the mathematical model for *CBF*.

For infants with *WG* < 30, *pCO*_*2*_ values averaged over all postnatal days were not statistically different for the risk and control groups (48.51 ± 11.45 mmHg vs. 46.75 ± 9.64 mmHg) and were above or close to the upper bound of normocapnia condition determined in different experimental studies as 45 mmHg (38) or 55 mmHg (39).

### Analysis of simulation results

#### Accounting of the germinal matrix

**Figure 4:**
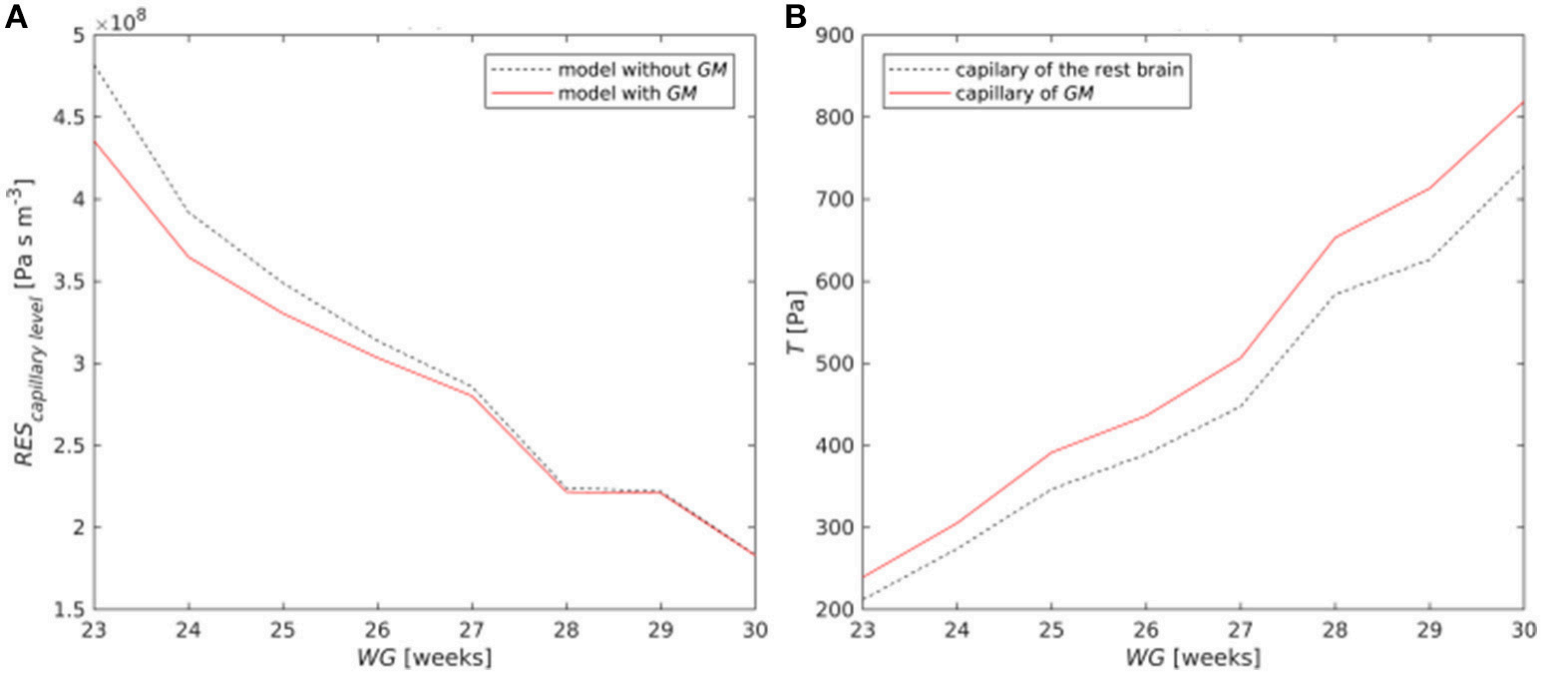
**(A)** Resistance of the capillary layer with and without *GM*; **(B)** Wall tension in a single capillary of the *GM* and in another area of the brain.

Inclusion of the *GM* in the model at the capillary level (*j* = *10)* changed the resistance of the whole layer (Figure 4A). The resistance of the whole capillary layer *RES*_10_ was reduced, especially for earlier *WG*. The average value of the resistance for infants with *WG* = 23 decreased from 4.8 × 10^8^ Pa s m^−3^ to 4.3 × 10^8^ Pa s m^−3^. The effect of this reduction was small (<1%) for global *CBF*, but it was considerable for blood flow in individual capillaries, especially for low *WG*. Thus, inclusion of the *GM* produced a decrease in blood flow in individual capillaries in other areas of the brain up to 13.5% for 23 *WG*, up to 9.7% for 24 *WG*, and to only 0.3% for 30 *WG*. Furthermore, the most relevant consequence of the presence of the *GM* was a 20% larger wall tension in capillaries of *GM* in comparison with that of the rest brain (Figure 4B), which was evaluated with Laplace's law (see section Accounting for the Presence of the Germinal Matrix).

#### Comparison of model based CBF and its reactivity to *pCO_*2*_* and MABP with experimental results

Because of the lack of clinical measurements of *CBF* for preterm infants of all gestational ages analyzed here, the comparison between our numerical results (Table 5) and experimental measurements available from the literature (Table 6) was made using values averaged over gestational ages. The reference experimental data were taken from intravenous Xenon-133 clearance measurements (10, 13, 14), computer-controlled pulsed Doppler measurements (9, 15), NIRS (10–12), and MRI ASL (17) techniques. Another promising modern non-invasive method for *CBF* measurements is diffuse correlation spectroscopy (DCS). However, DCS measured flow needs a calibration against a baseline *CBF* measured by another techniques (18–20). Since such data were not at our disposal, we did not compare calculated *CBF* with DCS measurements.

**Table 5:** Numerical values of *CBF* and its reactivity to *pCO*_2_ *(R*_*pC*_*O*__2__*)* and *MABP (R*_*MABP*_*)*.

***WG***	***IVH***	**Number of patients**	**Postnatal days**	***CBF* [ml min^−1^(100g)^−1^]**	***R_*pC*_*O*__2__*[% kPa^−1^]**	***R_*MABP*_*[% mmHg^−1^]**
23–30	No	129	1st	8.39 ± 3.72	8.09 ± 1.76	2.59 ± 1.45
			2nd	10.62 ± 5.71	28.05 ± 2.81	1.95 ± 1.21
			2nd-8th	12.07 ± 5.99	28.52 ± 2.86	1.98 ± 1.12
	With	136	1st	9.02 ± 4.45	8.18 ± 1.88	2.67 ± 1.40
			2nd	11.15 ± 7.08	28.20 ± 2.95	2.30 ± 1.20
			2nd-8th	12.99 ± 8.02	28.74 ± 2.92	2.22 ± 1.08
	All	265	1st	8.71 ± 4.1	8.14 ± 1.82	2.64 ± 1.42
			2nd	10.92 ± 6.52	28.14 ± 2.89	2.15 ± 1.22
			2nd-8th	12.56 ± 7.15	28.64 ± 2.89	2.11±.10

**Table 6:** Experimental values of *CBF* and its reactivity to *pCO*_2_ *(R*_*pCO*2_*)* and *MABP (R*_*MABP*_*)* taken from the literature (9–14, 17, 22, 27).

***IVH***	**Postnatal days**	***CBF* [ml min^−1^(100g)^−1^]**	***R_*pCO*2_*[% kPa^−1^]**	***R_*MABP*_*[% mmHg^−1^]**
No	1st	8.4 (2–12 h) (13)		
		10.2 (12–24 h) (13)		
	2nd	11.5 (13)		
		16.5 ± 2.1 (pre-treated) (12)		
		11.8 ± 1.2 (post-treated) (12)		
All	1st-2nd	14 ± 1 (hypotensive) (11)		
		19 ± 1 (normotensive) (11)		
	1st-4th	13.6 (hypotensive) (14)		1.9 (14)
		13.3 (normotensive) (14)		
	2nd-8th	12.51 (9)	32.7 (9)	1.0 (9)
		12.63 (10)	28.8 (22)	
			30 (27)	
	>7th	8.5 ± 6.1 (17)		

For patients without *IVH* model based *CBF* was equal to 8.39 ml min^−1^ (100 g)^−1^ for the first day of life and to 10.62 ml min^−1^ (100 g)^−1^ for the second day of life, which was in a good agreement with experimental values (13) of 8.4 ml min^−1^ (100 g)^−1^ for period 2–12 h, 10.2 ml min^−1^ (100g)^−1^ for period 12–24 h, and 11.5 ml min^−1^ (100 g)^−1^ for period 24–48 h.

During the period from the 2nd to the 8th day, estimated *CBF* for all patients increased up to 12.56 ml min^−1^ (100 g)^−1^, whilst Doppler measured experimental value (9) was 12.51 ml min^−1^ (100 g)^−1^, Xenon measured (10) values were equal to 12.63 ml min^−1^ (100 g)^−1^ and 13.3 ml min^−1^ (100 g)^−1^ for later postnatal days (14), and NIRS measured (11) values were equal to 14 ml min^−1^ (100 g)^−1^ for hypotensive and to 19 ml min^−1^ (100 g)^−1^ for normotensive preterm infants.

Numerically calculated reactivity of *CBF* to *pCO*_*2*_ for all patients was equal to 8.14% kPa^−1^ on the first day of life, but already on the second day, it increased up to 28.16% kPa^−1^, which agrees with reports on decreased *pCO*_*2*_ reactivity on the first day of life (15, 27). The average value computed over all patients for a time period from the 2nd to the 8th postnatal day was equal to *R*_*pCO*2_ = 28.64% kPa^−1^, which is lower than the reactivity (9) of 32.7% kPa^−1^, but is similar to the reactivity (22) of 28.8% kPa^−1^ and the reactivity (27) of 30% kPa^−1^. The numerically estimated mean value of *R*_*MABP*_ over all patients in the time period from the 2nd to the 8th day was equal to 2.11% mmHg^−1^, which is almost twice higher than the Doppler-measured (9) value of 1% mmHg^−1^, but agrees with the Xenon measured (14) one of 1.9% mmHg^−1^.

Variability of model based *CBF* values depending on three constant input parameters (intracranial pressure, *P*_*ic*_, baseline value $pCO_{2}^{norm}$, and dynamic viscosity of blood μ) was estimated. The response of *CBF* to ±10% variation of these parameters, averaged over all infants with gestational age from 23 *WG* to 30 *WG* and postnatal age from 2nd to 8th days, is presented in Table 7. The variation of *CBF* was around 1.5% for *P*_*ic*_, about 13% for $pCO_{2}^{norm}$, and ~10% for μ. However, all model based values of *CBF* were in the range from 11.03 to 14.18 ml min^−1^ (100 g)^−1^, which matched the experimentally measured range from 8.04 to 19 ml min^−1^ (100 g)^−1^ (Table 6).

**Table 7:** Effect of variations of input constant parameters on the model based *CBF*.

**Parameter**	**Variation: ±10%**	***CBF* [ml min^−1^ (100)^−1^]**
*P_*ic*_* [mmHg]	4.5	12.75 ± 7.22(+1.5%)
	5 (in model)	12.56 ± 7.15
	5.5	12.36 ± 7.07(-1.6%)
${\mathrm{pCO}}_{2}^{norm}$ [mmHg]	36	14.18 ± 7.88(+12.9%)
	40 (in model)	12.56 ± 7.15
	44	11.03 ± 6.46(-12.2%)
μ [10^−3^ Pa s]	2.61	13.85 ± 7.88(+10.3%)
	2.9 (in model)	12.56 ± 7.15
	3.19	11.49 ± 6.54(-8.5%)

#### Analysis of model based CBF and its reactivity to *pCO_*2*_* and MABP

**Figure 5:**
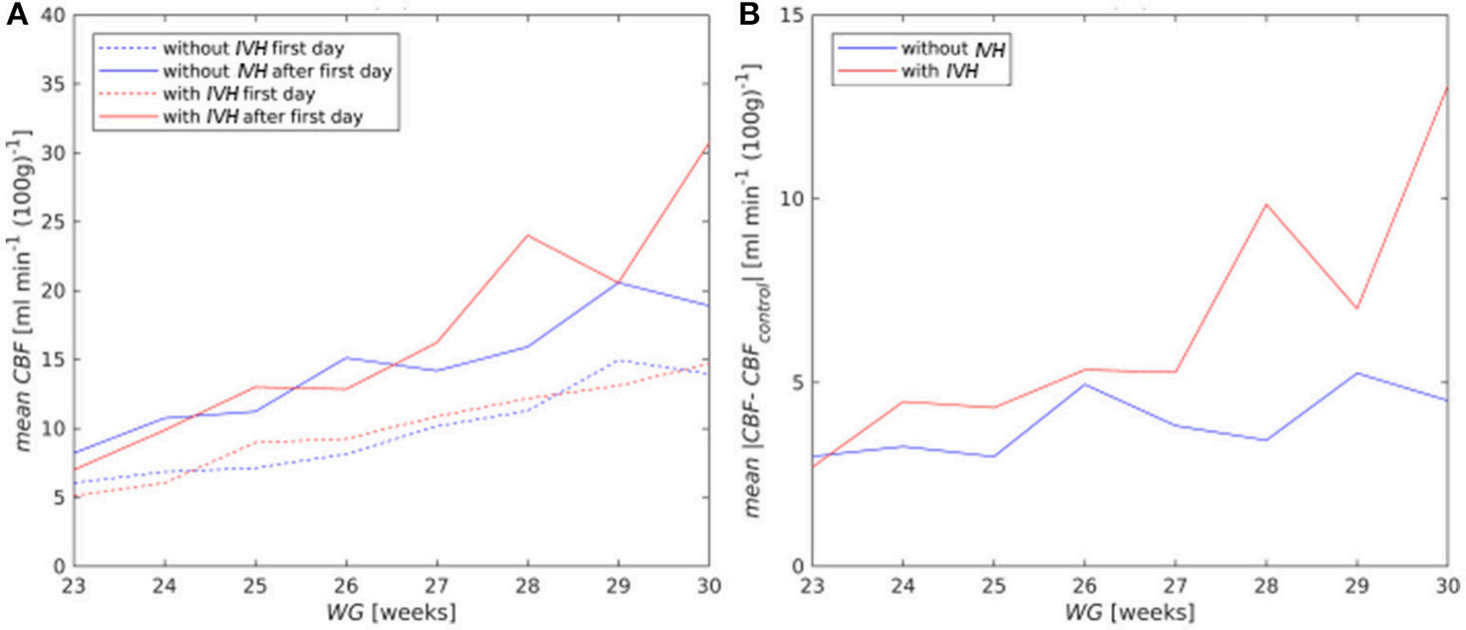
**(A)** Mean *CBF* for the first (dashed curves) and after the first day of life (solid curves); **(B)** mean absolute deviation of *CBF* from the mean value of the control group (*CBF*_*control*_) for all postnatal days.

Model based *CBF* values increased with gestational and postnatal ages for the both control and risk groups (Figure 5A). No statistically significant difference in mean *CBF* between the control and risk groups was observed. However, fluctuations of *CBF* around the mean value for the control group (Figure 5B) were larger for patients with *IVH* than without *IVH* (*p* = 0.065).

**Figure 6:**
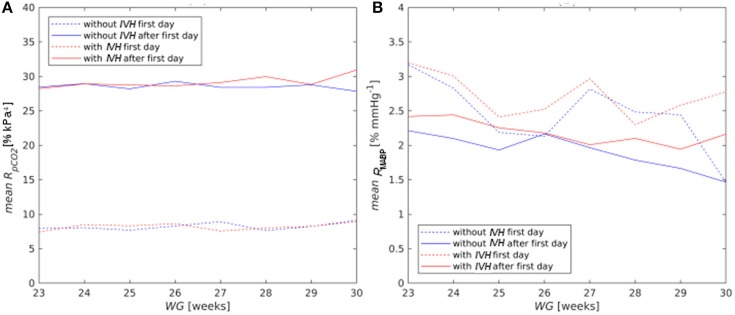
Mean reactivity of *CBF* on *pCO*_*2*_ **(A)** and *MABP* **(B)** for the first (dashed curves) and after the first day of life (solid curves).

On the first postnatal day, *CBF* reactivity to *pCO*_*2*_ (*R*_*pC*_*O*__2__) averaged over all patients was equal to 8.16% kPa^−1^, but already on the second day it increased almost to normal values both in the control group (28.07% kPa^−1^) and in the risk group (28.23% kPa^−1^). The *R*_*pC*_*O*__2__ value averaged over the time period from the 2nd to 8th postnatal days was equal to 28.52% kPa^−1^ for the control group and to 28.74% kPa^−1^for the risk group. No difference across gestational age and between the risk and control groups was observed (Figure 6A).

*CBF* reactivity to *MABP* (*R*_*MABP*_) was high for all newborns on the first day (2.64% mmHg^−1^) and decreased with postnatal and gestational age (Figure 6B), although it stayed always higher for the risk group than for the control one. The *R*_*MABP*_ value averaged over the time period from the 2nd to 8th postnatal days was higher (*p* = 0.02) for the risk group (2.22% mmHg^−1^) than that for the control one (1.98% mmHg^−1^).

## Discussion

The main purpose of the present work was (I) the adjustment of a mathematical model for the calculation of *CBF* to the peculiar characteristics of the immature cerebral circulation; (II) the comparison of model predictions with equivalent experimental data from the literature, and (III) a statistical analysis of model based *CBF* and its reactivity to *MABP* and *pCO*_*2*_ changes for different gestational and postnatal age of patients with and without *IVH*.

### Adjustment of the hierarchical cerebrovascular model to immature brain

A mathematical model for assessment of *CBF* (22) was adapted to cerebral hemodynamics of the immature brain by including a microvascular network describing the *GM* into the hierarchical cerebrovascular blood flow according to the gestational age of preterm infants. When adapting the model, some constant values of parameters (e.g., intracranial pressure *P*_*ic*_, baseline value $pCO_{2}^{norm}$ and dynamic viscosity of blood μ) were taken from the literature. Even if the literature sources suggest slightly different values of these parameters, our evaluation showed that the effect of a 10% variation of each of them preserved the model based *CBF* in the ranges reported in the literature.

Accounting for the *GM* in the modeling of the capillary level helped to understand specific features of immature brain. Since *GM* volume constitutes only up to 5% of the total brain volume, the effect of the *GM* on the global *CBF* was small (<1%), but it was noticeable at the capillary level. For a gestational age of 23 weeks, the numerically computed resistance of the capillary level decreased from 4.8 × 10^8^ Pa s m^−3^ to 4.3 × 10^8^ Pa s m^−3^ (Figure 4A) after accounting for the *GM*. This drop was in agreement with the mathematically predicted drop (42) from 8 × 10^8^ Pa s m^−3^ to 6.9 × 10^8^ Pa s m^−3^ [second value was calculated as (*RES*_*GM*_^−1^ + *RES*_*B*_^−1^)^−1^ with *RES*_*GM*_ = 51.5 × 10^8^ Pa s m^−3^ and *RES*_*B*_ = 8 × 10^8^ Pa s m^−3^, see Table 4 from the article (42)] in a 6-edge topology model of the capillary network including *GM*. Furthermore, accounting for the *GM* can change blood flow in individual vessels up to 13.5%. Additionally, the wall tension in capillaries of the *GM* was estimated to be 20% higher than that in the rest brain, which occurred due to the larger diameter of the capillary vessels of the *GM* in comparison with that of other brain regions. This may explain the fact that most hemorrhages originate in the *GM* (2). The observed increase of wall tension is significant in modeling biomechanical stresses in vessel walls and evaluation of hazard values of medical characteristics leading to vessel rupture and *IVH*.

### Comparison of numerical estimations with experimental measurements

Model based *CBF* and its reactivity to *MABP* and *pCO*_*2*_ was in a good agreement with experimental data from the literature (9–14, 17). Also larger fluctuations of computed *CBF* for infants with *IVH*, compared to those without *IVH*, were in accordance with strongly fluctuating *CBF* observed experimentally as a result of impaired cerebral autoregulation in preterm infants (7, 8, 43, 44).

A good agreement of the numerical results with the experimental measurements confirms the ability of the model to correctly describe effects of *MABP* and *pCO*_*2*_ on *CBF* in immature brain. Thus, the model developed can be applied for the analysis of clinical parameters which are critical for the occurrence of *IVH*. Hierarchical modeling of the cerebral vasculature enables us to calculate the pressure and estimate shear stresses in walls of *GM* vessels, where *IVH* usually originates.

Furthermore, in the absence of *CBF* measurements during clinical monitoring of preterm infants, numerically calculated extreme values and high fluctuations of *CBF* can be regarded as risk factors of *IVH*.

### Statistical analysis according to the gestational and postnatal age

An important advantage of the present study was its large sample size (3,439 clinical records), which allowed the analysis of baseline clinical parameters and numerical results obtained for infants with and without *IVH*. The evolution of the results according to gestational and postnatal ages could also be analyzed.

For infants of the risk group with *WG* < 25, strong hypotension was established from the first day and for those with *WG* < 28 from the second day on. However, an increase of mean *MABP* with gestational and postnatal ages was observed both for the control and risk groups.

Model based *CBF* increased with gestational and postnatal ages. Although absolute values of *CBF* were not significantly different between the risk and control groups, the fluctuations of *CBF* were significantly higher in the risk group for all gestational ages. That is, the occurrence of critical high (low) values of *CBF* and, therefore, of hemorrhages (ischemia), are more likely in the risk than in the control group.

The reactivity of *CBF* to *pCO*_*2*_ did not depend on gestational age, but the reactivity of *CBF* to *MABP* decreased with gestational and postnatal age, which means a stabilization of *CBF* behavior toward intact autoregulation.

Statistical analysis of clinically measured *pCO*_*2*_ showed no difference regarding the gestational age. Depending on the definition of the upper limit of normocapnia [45 mmHg (38) or 55 mmHg (39)] we found established or close to established hypercapnia values for both control and risk groups. Previous experimental studies (14, 39, 43) demonstrated that hypercapnia leads to vasodilation, which in turn increases the risk of *IVH* in preterm infants. However, in the present study, mean *pCO*_*2*_ was not statistically different in the risk and control groups. This observation suggests that hypercapnia alone cannot be responsible for the development of *IVH*. More likely, this occurs in combination with hypotension and/or strong fluctuating *CBF*.

## Limitations and further work

The model developed was calibrated using averaged values of *MABP* and *pCO*_*2*_ of the control group. Though processing of less cluttered data is easier and provides smoother results, this can withdraw some valid samples from the consideration. The model was parameterized using clinical data collected in two clinics, and its good predictive ability was demonstrated using experimental data from the literature. However, the model should additionally be validated by analyzing the *CBF* behavior in an independent clinical group.

The model accounts for the dependence of *CBF* on only two important clinical parameters, *MABP* and *pCO*_*2*_. They are, however, recognized as the main factors affecting *CBF* (27). A statistical analysis was done to evaluate the relation between *CBF, MABP, pCO*_*2*_, and the origin of *IVH*. The predictive power of the model with respect to the origin of *IVH* was not studied because the latter is multifactorial, and, therefore, an analysis of other risk factors affecting cerebral hemodynamics like, e.g., amount of thrombocytes, presence of inflammation, etc., is necessary. Such a study and an appropriate extension of the current model are planned.

Our mathematical simulation involves the whole cerebrovascular network. Based on a model of micropolar fluid flow in circular pipes (28), adjusted to blood reology (29, 30), we were able, to some extent, to take into consideration non-Newtonian characteristics of blood. However, the model does not account for spatial vessel structure and hematocrit distributions in microcirculation as described, e.g., in (45, 46). Future work will include accounting for red blood cells distributions and more realistic 3D topologies of microcirculatory networks using approaches like those described in (47). Additional improvements can also be achieved by using real data sets presenting spatial architectures of cerebral vessel trees (48), network topologies of the capillary system (42), effects of penetrating vessel occlusions (49), and other hematological parameters relevant for the description of immature brain of preterm infants.

The model developed has a significant potential for both research and clinical purposes. Regular monitoring of the *CBF* level and its fluctuations in preterm infants is important to assess impaired cerebral autoregulation leading to complications such as *IVH*. Although several techniques for measuring *CBF* are currently available, they are not yet part of clinical routine. Model based prediction of *CBF* values can provide complementary information to routine clinical measurements. The origin of *IVH* is multifactorial, and model based estimated *CBF* can be used as one of the independent variables in multivariate statistical analysis of risk factors of *IVH*.

An advantage of the hierarchical cerebrovascular model is the possibility to evaluate physical characteristics of the microcirculatory network from standard clinical measurements. The estimation of blood flow velocity and pressure in individual vessels enables the assessment of biomechanical stresses in walls of capillaries and determination of critical values of *MABP* and *pCO*_*2*_ beyond which the likelihood of disruption of *GM* vessels grows, and the probability of an hemorrhage increases.

## Conclusions

A hierarchical cerebrovascular model for the adult brain (22) was modified according to infants' gestational age by scaling the vessel number and size to brain weight and by accounting for the presence of the *GM* in the calculation of vascular resistance at the capillary level. Furthermore, values of clinically measured *MABP* were employed for the adaptation of an autoregulation model to preterm infants. Intact or impaired autoregulation was simulated as vascular response to changes in *MABP* and *pCO*_*2*_ and accounting for gestational and postnatal age. Additionally, a micropolar fluid model describing blood flow in vessels accounting for microrotations of red blood cells suspended in plasma was applied to the calculation of the hydraulic resistance for vessels of each hierarchical level. To the best of our knowledge, there is no other model of cerebral blood circulation in the immature brain which includes all these features. Model based values of *CBF* and its reactivity to changes in *MABP* and *pCO*_*2*_ showed a good agreement with equivalent experimental measurements described in the literature. Therefore, the developed model can be proposed as a complimentary tool for assessing *CBF* in preterm infants and for estimating the physical characteristics of the microvessel network from standard medical parameters, hazard for biomechanical failure of capillaries in the *GM* and origin of *IVH*.

## Data availability statement

The data underlying the study are available as Supplementary Table 1.

## Author contributions

RL: supervising; VT, NB, IS, and AK: model development; LE, ER-F, and UF-M: Data collection; IS, VT, and NB: numerical simulations; IS, VT, and AA-P: statistical analysis. Each author contributed important content during model development, data analysis and interpretation, manuscript drafting and revision. All authors approved the final version of the manuscript and agree to be accountable for the content of the work.

### Conflict of interest statement

The authors declare that the research was conducted in the absence of any commercial or financial relationships that could be construed as a potential conflict of interest.

## Abbreviations

IVH: intraventricular hemorrhage
ICP: intracranial pressure
WG: weeks of gestation
GM: germinal matrix
MABP: mean arterial blood pressure
*pCO*_*2*_: carbon dioxide pressure
CBF: cerebral blood flow.
